# Three-month functional outcomes of acute ischemic stroke in patients with chronic renal function impairment

**DOI:** 10.1371/journal.pone.0323995

**Published:** 2025-05-29

**Authors:** Dina Hemmati, Negin Eissazade, Shayan Eghdami, Zahra Mirzaasgari, Atefeh Amouzegar

**Affiliations:** 1 Students Research Committee, School of Medicine, Iran University of Medical Sciences, Tehran, Iran; 2 Cellular and Molecular Research Center, Iran University of Medical Sciences, Tehran, Iran; 3 Brain and Cognition Clinic, Institute for Cognitive Sciences Studies, Tehran, Iran; 4 Department of Neurology, School of Medicine, Iran University of Medical Sciences, Tehran, Iran; 5 Firoozgar Clinical Research Development Center (FCRDC), Iran University of Medical Sciences, Tehran, Iran; 6 Department of Nephrology, Endocrine Research Center, Institute of Endocrinology and Metabolism, Iran University of Medical Sciences (IUMS), Tehran, Iran; Universidade Federal de Mato Grosso do Sul, BRAZIL

## Abstract

**Background:**

Chronic renal function impairment (RFI) increases the risk of acute ischemic stroke (AIS), greater stroke severity, and post-stroke disability and mortality.

**Objective:**

We aimed to assess the three-month functional outcomes of AIS in patients with chronic RFI.

**Methods:**

In this prospective study, stroke severity was assessed using the National Institutes of Health Stroke Scale (NIHSS), and functional outcomes were assessed using the modified Rankin Scale (mRS). Outcomes were analyzed based on estimated glomerular filtration rate (eGFR).

**Results:**

Among the 205 patients included (median age: 70 years, IQR: 61–82), 123 (60%) were male. Patients with lower eGFR had higher NIHSS scores (more severe strokes) and higher mRS scores at both baseline and three months (poorer functional outcomes). Based on a logistic regression model adjusted for confounding factors, higher eGFR was significantly associated with good three-month functional outcomes (adjusted OR = 2.634, 95% CI [1.207, 5.748], p = 0.015).

**Conclusions:**

Chronic RFI is associated with more severe strokes and poorer baseline and three-month functional outcomes after AIS. Future research should explore targeted management strategies to improve post-stroke recovery in this population.

## Introduction

Acute ischemic stroke (AIS) is a leading cause of death and disability worldwide [1], with outcomes largely influenced by underlying comorbidities [2]. Established risk factors for AIS—such as old age, diabetes, hypertension, dyslipidemia, and cardiovascular disease [3–5] —are also major contributors to chronic renal function impairment (RFI) [6,7].

Beyond traditional risk factors, AIS and chronic RFI share non-traditional pathophysiological mechanisms, including systemic inflammation, oxidative stress, and endothelial dysfunction, all of which can impair cerebral blood flow regulation and worsen stroke severity. Furthermore, the accumulation of toxins and metabolic waste in patients with chronic RFI promotes a pro-thrombotic state, further exacerbating stroke damage [5,7,8]. However, the precise role of these factors remains unclear.

Patients with chronic RFI face a significantly higher risk of stroke (5–30 times), greater stroke severity, and a higher likelihood of post-stroke complications such as functional disability, kidney failure and mortality [5,9,10]. Moreover, chronic RFI often remains asymptomatic until advanced stages [11], thereby increasing the risk of morbidity and mortality. Furthermore, the impact of chronic RFI on AIS outcomes—both short- and long-term—varies by stroke subtype, race, and other vascular risk factors [2,5].

Previous studies have reported inconsistent findings regarding the short-term outcomes of AIS in patients with RFI [12–17]. Given the potential influence of chronic RFI on post-stroke recovery, we aimed to assess its association with stroke severity and three-month functional outcomes in patients with AIS. Specifically, we sought to answer the research question: Does chronic RFI affect the three-month functional outcomes in AIS patients?

## Materials and methods

### Study design and participants

This prospective study was designed to assess the three-month functional outcomes of AIS in patients with chronic RFI. It was conducted at Firoozgar Hospital, a stroke referral center (affiliated with Iran University of Medical Sciences, Tehran, Iran), between March 1, 2022 and January 1, 2023.

The study protocol was approved by the Ethics Committee of Iran University of Medical Sciences Institutional Review Board (IR.IUMS.REC.1400.1001). Only the researchers had access to the data. Written informed consent was obtained from all participants, and all methods were carried out in accordance with the relevant ethical guidelines.

Inclusion criteria were: (1) AIS confirmed by brain computed tomography (CT) scan or magnetic resonance imaging (MRI); and (2) the presence of clinical signs and symptoms consistent with AIS (e.g., sudden-onset hemiparesis, speech disturbances, facial droop, ataxia, or visual impairment) persisting for more than 24 hours. Patients were excluded if they had elevated serum creatinine levels due to causes other than chronic RFI (e.g., acute kidney injury, renal stones), or if they had end-stage renal disease requiring dialysis or kidney transplantation.

### Data collection and tools

Data were collected prospectively from the patients’ electronic medical records and structured follow-up interviews. Baseline information included demographics, comorbidities (e.g., hypertension, diabetes mellitus, dyslipidemia, and atrial fibrillation), neuroimaging findings, and serum creatinine levels. Renal function was assessed using estimated Glomerular Filtration Rate (eGFR), which was estimated based on the 2021 Chronic Kidney Disease Epidemiology Collaboration (CKD-EPI) equation [18]. Patients were categorized into two groups based on an eGFR threshold of 60 mL/min/1.73m² [18].

Stroke severity upon admission was assessed using the National Institutes of Health Stroke Scale (NIHSS) [19], and post-stroke functional outcomes were evaluated with the modified Rankin Scale (mRS) [20]. Three months after stroke onset, functional outcomes were obtained via follow-up phone interviews using the mRS.

The NIHSS consists of 11 items assessing specific neurological functions, each scored from 0 to 4. These items include level of consciousness, horizontal eye movement, visual field testing, facial palsy, motor function of the arm and leg, limb ataxia, sensory function, speech, language, extinction, and inattention. Stroke severity is classified as minor (1–4), moderate (5–15), moderate-to-severe (16–20), and severe (21–42) based on the total score [19].

The mRS was used to categorized functional outcomes as good (0–2), poor (3–5), and death (6). mRS scores are assigned as follows: 0 – no symptoms; 1 – no significant disability (able to perform usual activities despite some symptoms); 2 – slight disability (unable to perform all previous activities but able to manage personal affairs without assistance); 3 – moderate disability (requiring some help but able to walk unassisted); 4 – moderately severe disability (unable to walk or attend to personal needs without assistance); 5 – severe disability (bedridden, incontinent, and requiring constant care); and 6 – death [20].

### Sample size and statistical analysis

The required sample size was calculated as 105 patients per group, based on a power level of 80% (1-β), an α-error of 5%, and an effect size of 0.23.

The Kolmogorov-Smirnov test was used to assess data normality. Continuous variables are presented as median with interquartile range (IQR), while categorical variables are expressed as frequencies and percentages. Categorical variables were compared using the Chi-square test, and non-normally distributed continuous variables were compared using the Mann-Whitney U test. A logistic regression model was employed to adjust for confounding factors and to assess the independent association between eGFR and three-month functional outcomes. All statistical analyses were performed using the IBM SPSS software version 27, with statistical significance set at p < 0.05.

## Results and discussion

A total of 205 patients, with a median age of 70 years (IQR: 61–82), were included, of whom 123 (60%) were male. Lower eGFR was significantly associated with older age (U = 25.35, p < 0.001), higher NIHSS scores and higher mRS scores at both baseline and three months (Table 1).

**Table 1:** Demographic and clinical characteristics by eGFR Group.

	eGFR ≥ 60 (N = 103)	eGFR < 60 (N = 102)	Pearson Chi-Square (P-value)
Gender (male)	69 (67%)	54 (52.9%)	4.21 (0.04)
Age (median, IQR)	64 (56–71)	75 (67–84)	
Prior ischemic stroke	13 (12.6%)	24 (23.5%)	4.12 (0.04)
Comorbidities			
Hypertension	61 (59.2%)	74 (72.5%)	4.05 (0.04)
Diabetes mellitus	26 (25.2%)	44 (43.1%)	7.3 (0.007)
Dyslipidemia	20 (19.4%)	37 (36.3%)	7.25 (0.007)
Atrial fibrillation	16 (15.5%)	37 (36.3%)	11.5 (< 0.001)
Heart failure	19 (18.4%)	29 (28.4%)	2.85 (0.09)
Ischemic heart disease	26 (25.2%)	32 (31.4%)	0.95 (0.33)
Medications			
Antiplatelets	30 (29.1%)	42 (41.2%)	3.27 (0.07)
Anticoagulants	6 (5.8%)	14 (13.7%)	3.63 (0.06)
Treatment modality			7.95 (0.34)
rtPA	17 (16.5%)	11 (10.8%)	
Dual antiplatelet therapy	67 (65%)	69 (67.6%)	
Revascularization	4 (3.9%)	3 (2.9%)	
Hemorrhagic transformation	4 (3.9%)	–	0.33 (0.57)
NIHSS scores^1^			18.4 (< 0.001)
Minor	48 (46.6%)	20 (19.6%)	
Moderate	41 (39.8%)	64 (62.7%)	
Moderate to severe	13 (12.6%)	14 (13.7%)	
Severe	1 (1.0%)	4 (3.9%)	
Baseline mRS scores^2^			6.8 (0.009)
Good	85 (82.5%)	68 (66.7%)	
Poor	18 (17.5%)	34 (33.3%)	
Three-month mRS scores			24.62 (< 0.001)
Good	59 (57.3%)	24 (23.5%)	
Poor	19 (18.4%)	29 (28.4%)	
Expired	25 (24.3%)	49 (48.0%)	

**eGFR:** Estimated Glomerular Filtration Rate; **IQR:** Interquartile Range; **mRS:** modified Rankin Scale; **NIHSS:** National Institutes of Health Stroke Scale; **rtPA:** Recombinant tissue plasminogen activators.

1. NIHSS scores: minor (1–4); moderate (5–15); moderate-to-severe (16–20); and severe (21–42).

2. mRS scores: good (scores 0–2); poor (scores 3–5); and death (score 6).

Based on a logistic regression model adjusted for confounding factors, higher eGFR was significantly associated with good three-month functional outcomes (adjusted OR = 2.634, 95% CI [1.207, 5.748], p = 0.015).

Our findings indicated that lower eGFR is significantly associated with poorer three-month functional outcomes, supporting previous research that links chronic RFI with adverse stroke outcomes [7,17,21,22]. Specifically, patients with an eGFR < 60 mL/min/1.73 m demonstrated worse recovery compared to those with normal renal function, reinforcing the idea that chronic RFI serves as a critical prognostic factor for AIS recovery.

While chronic RFI has been previously linked to impaired cerebral autoregulation and an increased risk of hemorrhagic transformation, our study did not find a significant correlation between low eGFR and hemorrhagic transformation, possibly due to our limited sample size and the exclusion of patients with end-stage renal disease.

This study has several limitations that should be considered when interpreting the results. First, the sample size was relatively small, particularly within subgroups. Second, the study was conducted at a single center in Tehran, Iran, which may limit the generalizability of the findings to other populations. Additionally, eGFR was used as the sole measure of renal function, which may not have fully captured the complexity of renal impairment. While patients were advised to seek rehabilitation based on clinical recommendations, they were not systematically referred for physiotherapy sessions, and we did not have access to comprehensive data on rehabilitation participation. Another limitation is the reliance on follow-up phone calls for outcome assessment, without in-person clinical evaluations. This may have affected the reliability of certain outcomes that require objective neurological examination. Furthermore, despite controlling for confounders, unmeasured factors, such as lifestyle behaviors or genetic predispositions, may still have influenced the outcomes.

## Conclusions

Lower eGFR predicts poorer three-month functional outcomes of AIS in patients with RFI, highlighting the importance of renal function as a prognostic factor in stroke recovery. Further research with larger, multi-center cohorts and longer follow-up periods is necessary to better understand the role of renal dysfunction in stroke outcomes.

## Supporting information

S1 Data This file includes the raw data used to generate the study’s results. (XLSX)
